# Supplementary material for: Active learning streamlines development of high performance catalysts for higher alcohol synthesis
Source: Nat Commun. 2024 Jul 11;15:5844. doi: 10.1038/s41467-024-50215-1 (PMC11239856; doi:10.1038/s41467-024-50215-1)
Supplement: Supplementary file 1 — Supplementary Information [file 41467_2024_50215_MOESM1_ESM.pdf]

Active learning streamlines development of high performance catalysts for higher alcohol synthesis

Manu Suvarna†, Tangsheng Zou†, Sok Ho Chong, Yuzhen Ge, Antonio J. Martín, and Javier Pérez-Ramírez*

Institute for Chemical and Bioengineering, Department of Chemistry and Applied Biosciences, ETH Zurich, Vladimir-Prelog-Weg 1, 8093 Zurich, Switzerland.

† Equal contribution.

* Corresponding author. E-mail: jpr@chem.ethz.ch

Table of contents

Supplementary Notes…………………………………………………………………………...2

Supplementary Figures………………………………………………………………………..10

Supplementary Tables ……………………………………………………………….........25

Supplementary References ………………………………………………………………..58

Supplementary Notes

Supplementary Note 1 | Determining the chemical and parametric space

The composition of $Fe_xCo_yCu_zZr_a$ catalysts, with molar Fe, Co, Cu, and Zr contents denoted by x, y, z, and a, respectively, considered 1 mol% increments as the minimum step for each element with $x + y + z + a = 100$, resulting in 176,851 combinatorial possibilities and is referred to as the chemical space. In the case of reaction conditions, T spanned from 523 to 593 K with increments of 1 K, H_2:CO from 1.0 to 3.0 with increments of 0.1, and *GHSV* from 10,000 to 100,000 $cm^3\ h^{-1}\ g_{cat}^{-1}$ with increments of 5,000 $cm^3\ h^{-1}\ g_{cat}^{-1}$, resulting in 28,000 potential combinations and is referred to as the parametric space. The combination of the chemical and parametric spaces resulted in *ca.* $5*10^9$ combinations of potential catalyst composition and reaction conditions (176,851*28,000 = 4,951,828,000).

Supplementary Note 2 | Curation of seed data for Phase 1

The primary objective of Phase 1 was to map the space-time yield of higher alcohols (STY_{HA}) throughout the chemical space. The first step consisted on training the Gaussian process and Bayesian optimization (GP-BO) algorithm using the molar compositions and STY_{HA} from the Fe_xCo_yZr, $Fe_xCu_zZr_a$, and Co_yCu_zZr catalyst families, where Fe, Cu, and Co contents ranged from 1 to 100 mol% for up to two of the three elements, and Zr from 0 to 50 mol%.[1] Reaction conditions were maintained for all experiments (H_2:CO= 2.0, T = 533 K, P = 50 bar, and $GHSV$ = 24,000 $cm^3\ h^{-1}\ g_{cat}^{-1}$). This dataset, denoted as the seed dataset, contained 31 datapoints, each representing a unique catalyst formulation to develop Cycle 1 of Phase 1. The compositional information of seed catalysts was encoded assigning a zero value to the absent element(s) in a particular formulation to assure compatibility with the model architecture. For example, $Fe_{79}Cu_{10}Zr_{11}$ was denoted as $Fe_{79}Cu_0Co_{10}Zr_{11}$.[2,3] Seed catalysts are listed in **Supplementary Table 1** and compositional constraints listed in **Supplementary Table 2**.

Supplementary Note 3 | Curation of seed data for Phase 2

The primary objective of Phase 2 was to map STY_{HA} throughout the chemical and parametric space. Since the reaction conditions in Phase 1 were fixed (H_2:CO= 2.0, T = 533 K, P = 50 bar, and $GHSV$ = 24,000 cm^3 h^{-1} g_{cat}^{-1}), predictions in different areas of the parametric space would encounter the limitation of stationary data, *i.e.*, the intrinsic limitation of machine learning algorithms to extrapolate parameters kept constant in the training dataset. To counter this challenge, a set of selected catalysts synthesized in Phase 1 was evaluated at varying H_2:CO, T, and $GHSV$ with P = 50 bar for a total of 20 data points, denoted as seed experiments for Phase 2 (**Supplementary Table 12**). Values of STY_{HA} from the 20 seed experiments for Phase 2 along with the 30 experiments performed through the 5 cycles of Phase 1 were used to train the GP-BO algorithm and initiate Cycle 1 of Phase 2.

Supplementary Note 4 | Associated side reactions and operational considerations

HAS from CO hydrogenation (Equation 1) is subject to potential side reactions that may largely impact S_{HA}.[4,5] They include products such as carbon dioxide, methane, alkanes, alkenes, ketones, aldehydes, esters, and other oxygenates, among which carbon dioxide and methane exhibit the lowest economical value. The water-gas shift (WGS, Equation 2) reaction produces carbon dioxide and is typically catalyzed by copper. Methanation (Equation 3) is highly exothermic, presents a high H_2 consumption per mole of carbon and is commonly performed over cobalt catalysts.

$$n\mathrm{CO} + 2n\mathrm{H_2} \rightleftharpoons \mathrm{C}_n\mathrm{H}_{2n+1}\mathrm{OH} + (n - 1)\mathrm{H_2O} \quad (1)$$

$$\mathrm{CO} + \mathrm{H_2O} \rightleftharpoons \mathrm{CO_2} + \mathrm{H_2} \quad (2)$$

$$\mathrm{CO} + 3\mathrm{H_2} \rightleftharpoons \mathrm{CH_4} + \mathrm{H_2O} \quad (3)$$

The direct synthesis of higher alcohols typically requires H_2:CO = 1-2.[6] The WGS reaction is thus desirable for lower H_2:CO< 1 by generating H_2 and detrimental for H_2:CO> 2 for the same reason. A thermodynamic analysis of the reaction with H_2:CO = 2 and P = 30-50 bar reveals that hydrogen and water concentrations decrease with T, while those of the reactants increase. This study suggests optimal temperatures below 623 K, with preferred values in the range 553-593 K.[4,5] *GHSV* is related to contact time over the catalyst, with increased *GHSV* typically leading to greater higher alcohols productivity at the expense of CO conversion when the reaction is under kinetic control, while S_{HA} further benefits from increased *GHSV* when the reaction is mass transfer-controlled.[5,6]

Supplementary Note 5 | Limitations of the active learning framework

While the efficacy of active learning in finding catalytic systems optimizing multiple performance metrics for HAS was demonstrated, it is crucial to consider limitations defining its applicability.[7,8] This strategy is suitable for metrics sensitive to optimization, like STY_{HA} in this study, but is not of value for those intrinsically constrained to a narrow range. A prominent example is the mentioned relative insensitivity of product distribution we observed in FeCoCuZr catalysts, resulting in S_{HA} = 10-16% irrespective of catalyst composition and reaction conditions (**Supplementary Figure 8**). Furthermore, while the standardized synthesis method for all compositions minimizes architectural variations for improved consistency, this model does not consider structural features such as surface facets, defects, morphology, *etc.,* which are known to influence performance. It also lacks geometric or electronic descriptors, as their incorporation is challenging due to either the unavailability of readily accessible data or the necessity for resource-intensive characterization experiments and theoretical simulations. The inclusion of such physico-chemical information might improve predictive accuracy at the expense of time taken per cycle, reducing agility in the overall implementation.

Supplementary Note 6 | Estimated environmental and economic savings

Greenhouse gas emissions (in terms of CO_2 equivalents) and operating expenditure (in US$) were selected as metrics representing environmental footprint and economic costs, respectively. To estimate the size of a full traditional experimental program, we note that practical mapping of chemical and parametric spaces could require anywhere from hundreds to thousands of screening experiments. Based on representative studies of multicomponent catalysts for various chemical transformations,[3,9–12] we assume that a traditional program would involve an initial synthesis and screening of 400 multimetallic catalysts, and *ca.* 20 selected catalysts would be evaluated at 80 reaction conditions each, resulting in a total of 2000 catalytic tests, in order to obtain the outcomes in Phases 2 and 3 of the active learning program with similar precision in compositions and reaction conditions. Activities associated with both the traditional and active learning programs were classified into modeling, synthesis, testing, and data processing categories, and further broken down in terms of resources (chemical precursors, reactant gases, electricity) and personnel costs (**Supplementary Table 27**). The total number of work-days involved in each program represents the sum of personnel activity hours assuming eight-hour work days.

The environmental impact values of chemicals, gases, and electricity were obtained from premise v1.5.8[13] based on Ecoinvent v3.8.[14] The inventories in premise were derived from the results obtained using the IMAGE Integrated Assessment Model[15] for the year 2020. The environmental impact of the metal nitrates used were estimated using the value for the corresponding metal. Cost estimates for chemical reagents and gases used were based on their actual purchase costs in March 2023 at ETH Zurich, Switzerland. Cost estimates for electricity were obtained from June 2023 prices for business users based on online reports.[16] Cost estimates for personnel were based on average PhD salaries based on online reports.[17] These values are tabulated in **Supplementary Table 28**. Results are listed in **Supplementary Table 29**.

Supplementary Note 7 | Model architecture and hyperparameter tuning

The active learning model utilizes a Gaussian process regressor as the surrogate model implemented using the GPflow library. The objective of the Gaussian process algorithm was to predict STY_{HA} based on catalyst composition (Fe, Co, Cu, and Zr), *i.e.,* four inputs in Phase 1, or catalyst composition and reaction conditions (Fe, Co, Cu, Zr, H_2:CO, *T*, *GHSV*), *i.e.,* seven inputs in Phases 2 and 3. A key aspect to the model building process was the selection of the kernel function and its tuning to capture the relationships between the input and target variables. We selected the Squared Exponential kernel, with 4 lengthscales (hyperparameters) in Phase 1, and 7 distinct lengthscales in Phase 2 and 3, where each lengthscale corresponds to one of the input features. During each cycle of the active learning process, the hyperparameters of the Gaussian process model were tuned to achieve the desired predictive accuracy. In all the cases, the lengthscales were initialized with a default value of 0.1, and optimized using the SciPy optimizer, to minimize the negative log likelihood under a constant variance of 1.0. This methodology enabled automated selection of kernel lengthscales ensuring optimal model performance. The optimal lengthscale values for all cycles across Phases 1-3 are listed in (**Supplementary Table 30**).

Bayesian optimization was performed using the Trieste package as it contained necessary libraries including surrogate model selection and relevant acquisition functions. For single-objective optimization in Phases 1 and 2, we used a combination of expected improvement (EI), and predictive variance (PV) acquisition functions to maximize STY_{HA}. More specifically, during each cycle of Phase 1, two lists of 30 candidates each were generated separately using the EI and PV functions. The combined set of 60 recommended catalysts from the above steps were manually analyzed and 6 catalysts were selected for experimentation based on user-defined criteria (see **Methods**, Implementing the active learning loop). A similar procedure was adopted for Phase 2, as well as Phase 3, except that the expected hypervolume improvement (EHVI) acquisition function was used for multi-objective optimization.

Supplementary Note 8 | Evaluating model reliability *via* stratified cross-validation

Performing cross-validation on data generated from an active learning loop can be challenging due to the nature of the data generation process. In an active learning loop, the data points are not randomly sampled from a fixed dataset but are instead sequentially chosen based on the predictions of the surrogate model (Gaussian process regressor) and the recommendations of the Bayesian optimizer. Specifically, based on the points sampled either from predictive variance or expected improvement acquisition functions of the Bayesian optimizer, the dataset might have highly diverse or extremely biased datapoints, respectively. As such, traditional *k*-cross-validation techniques may not be directly applicable and could underestimate or overestimate the model reliability.

Stratified cross-validation addresses these challenges by preserving the data distribution in each fold, ensuring representative subsets for training and testing. In the context of active learning, this helps maintain the diversity of the dataset across folds, preventing biases introduced by the iterative selection process. In this study, we employ stratified cross-validation to evaluate model prediction reliability. After the completion of a particular Phase, we first fit the Gaussian process regressor to the entire dataset and evaluate the model performance in terms of R^2 and *MAPE*. Subsequently, we perform stratified cross-validation with *n*-folds = 6 and compare the accuracy metrics with the evaluation of the entire dataset (**Supplementary Table 31**). The results depict a significant deviation in Phase 1 as the *MAPE* during cross-validation is more than doubled. Although stratified cross-validation balances the data distribution, a significant portion of the datapoints originated from predictive variance (exploration) which could possibly explain this behavior. By Phases 2 and 3, the active learning was mostly steered towards exploitation in order to maximize the STY_{HA}, introducing less diversity into the dataset, allowing the model the learn and generalize better as observed by the close agreement between the R^2 and *MAPE* of the full dataset and during cross-validation.

Supplementary Figures

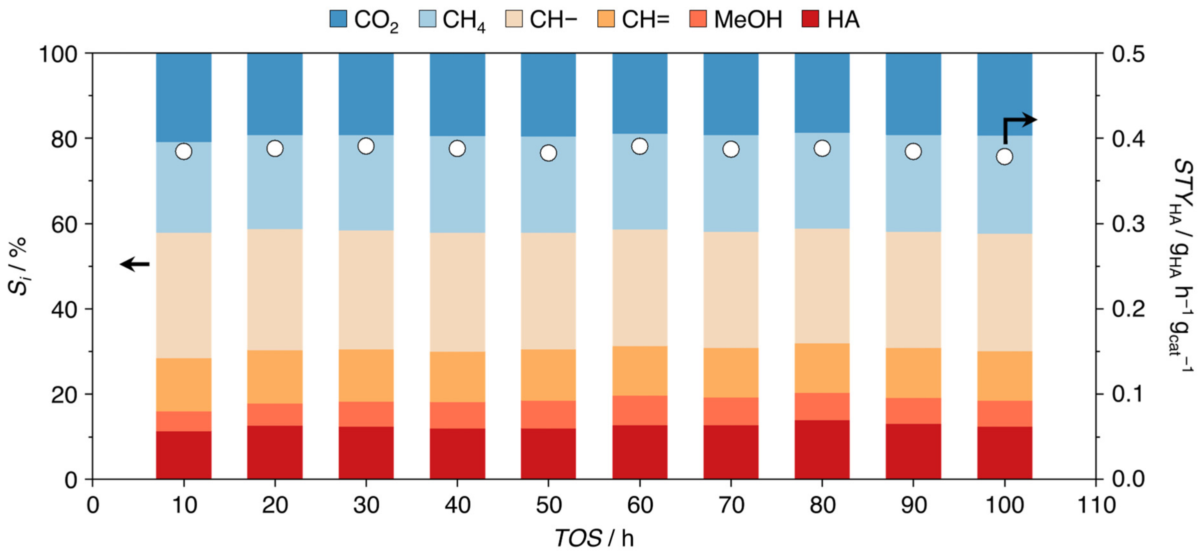

Supplementary Figure 1 | Temporal evolution of catalytic performance for the catalyst attaining the highest STY_{HA} in Phase 1 ($Fe_{69}Co_{12}Cu_{10}Zr_9$). Reaction conditions: H_2:CO = 2.0, T = 533 K, P = 50 bar, and $GHSV$ = 24,000 cm^3 h^{-1} g_{cat}^{-1}.

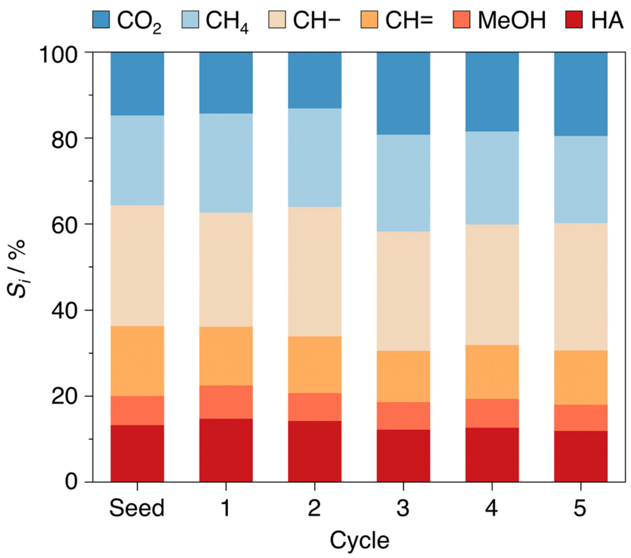

Supplementary Figure 2 | Product distributions for FeCoCuZr catalysts attaining the highest STY_{HA} in each active learning cycle of Phase 1 as detailed in **Supplementary Tables 3-8**. Reaction conditions: H_2:CO = 2.0, T = 533 K, P = 50 bar, and $GHSV$ = 24,000 cm^3 h^{-1} g_{cat}^{-1}.

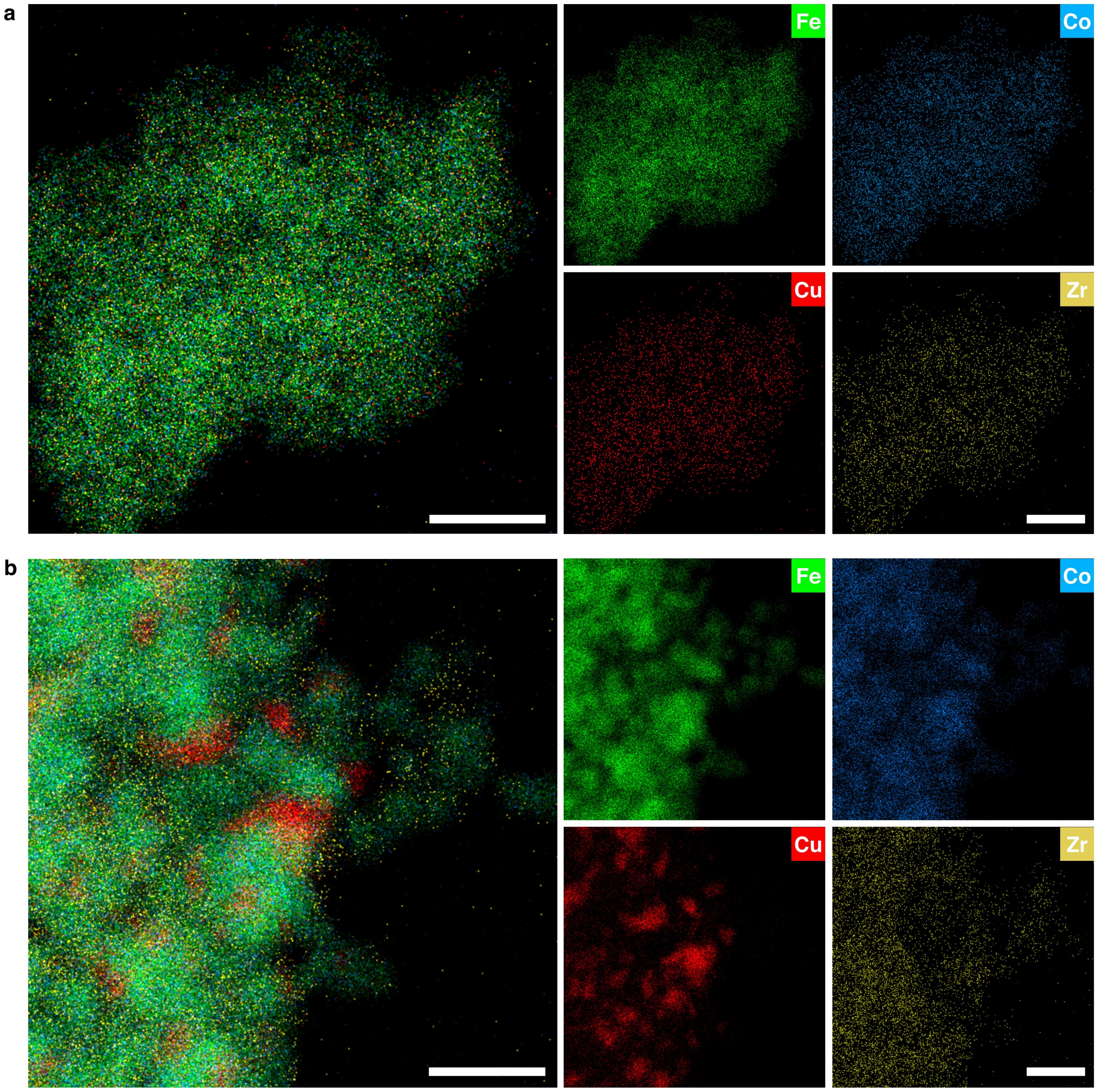

Supplementary Figure 3 | ** STEM-EDX elemental maps of $Fe_{69}Co_{12}Cu_{10}Zr_9$ **a) in fresh form and **b**) after 24 h on stream. Scale bars: 50 nm. Reaction conditions: H_2:CO= 2.0, T = 533 K, P = 50 bar, and $GHSV$ = 24,000 $cm^3\ h^{-1}\ g_{cat}^{-1}$.

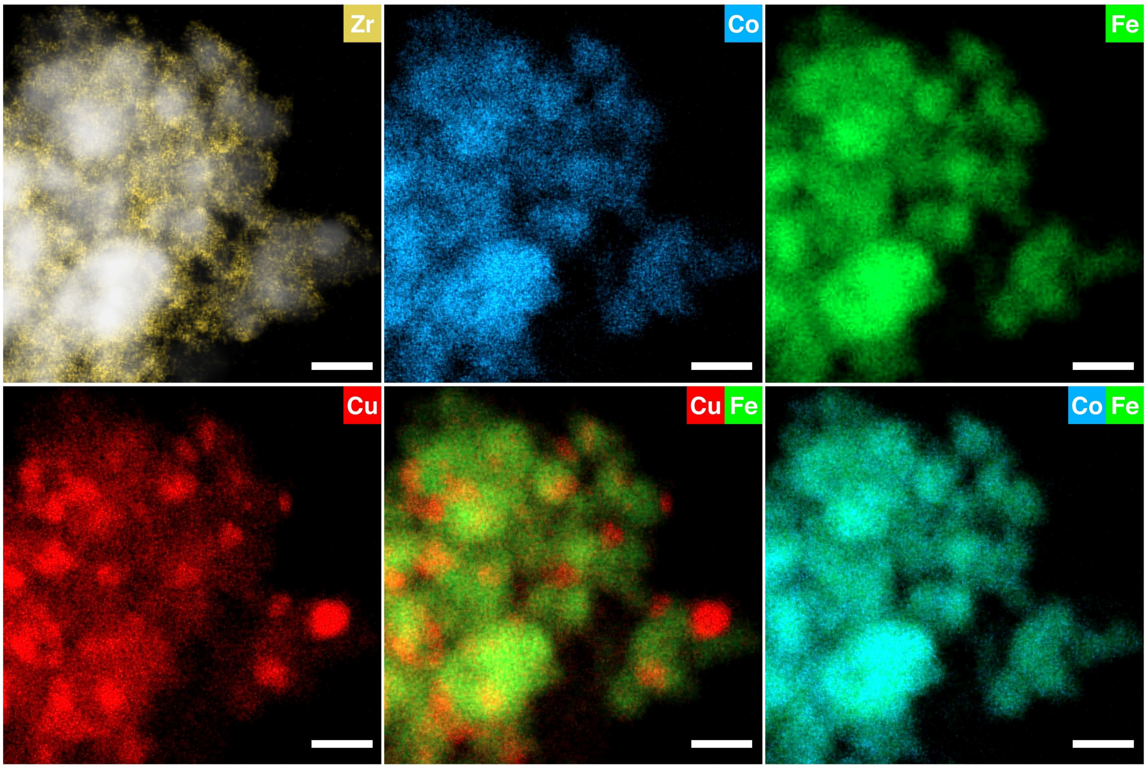

Supplementary Figure 4 | HAADF-STEM image and corresponding EDX maps of $Fe_{69}Co_{12}Cu_{10}Zr_9$ after 100 h on stream. Scale bars: 20 nm. Reaction conditions: H_2:CO= 2.0, T = 533 K, P = 50 bar, and $GHSV$ = 24,000 $cm^3\ h^{-1}\ g_{cat}^{-1}$.

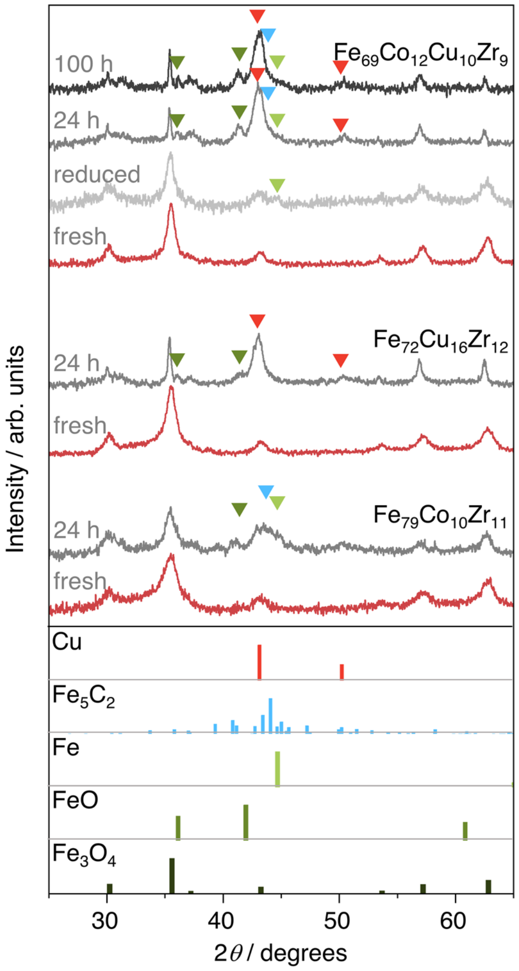

Supplementary Figure 5 | XRD patterns of the FeCoCuZr catalyst identified in Phase 1 and seed FeCuZr and FeCoZr catalysts attaining the highest STY_{HA} in their respective categories. Catalysts as calcined (red) and after various stages of reaction (grey) are shown. Reaction conditions: H_2:CO = 2.0, T = 533 K, P = 50 bar, and $GHSV$ = 24,000 cm^3 h^{-1} g_{cat}^{-1}.

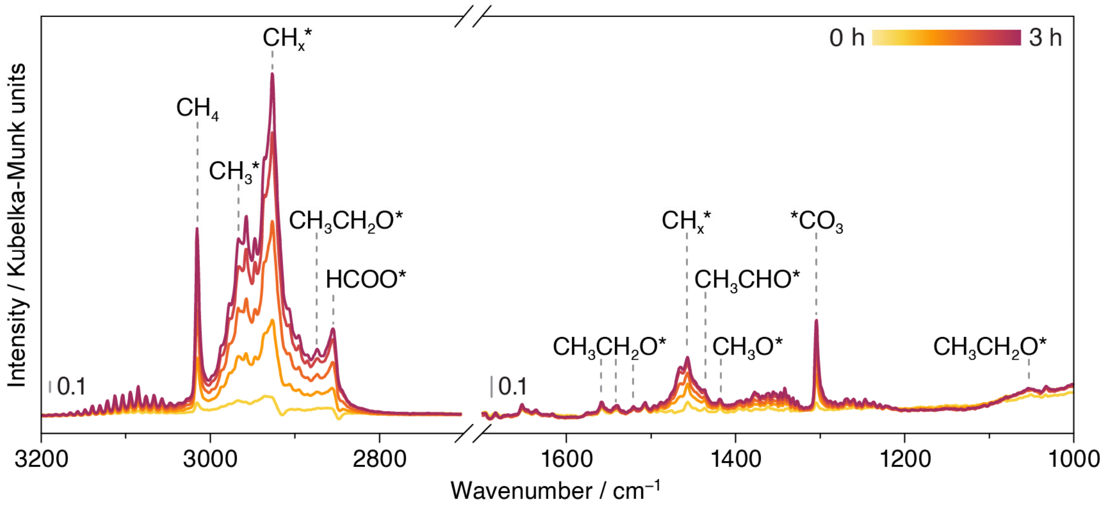

Supplementary Figure 6 | *In situ* DRIFT spectra for the $Fe_{69}Co_{12}Cu_{10}Zr_9$ catalyst. Reaction conditions: H_2:CO = 2.0, T = 533 K, P = 20 bar, m_{cat} = 10 mg, F_T = 7.5 cm^3 min^{-1}, and dwell time = 180 min. Peaks assigned to surface intermediates and products are based on literature references.[18,19]

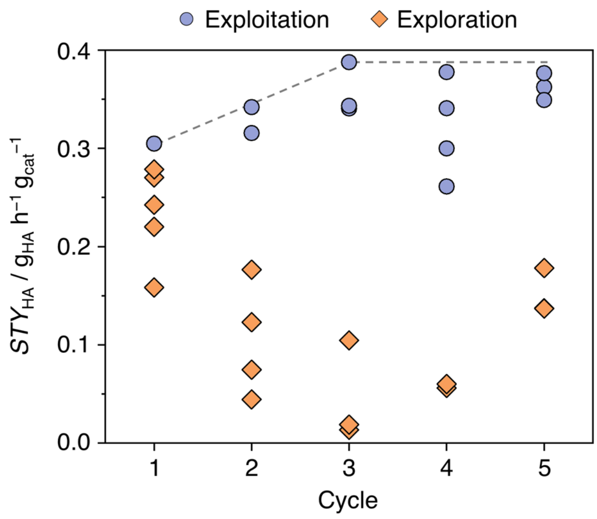

Supplementary Figure 7 | STY_{HA} obtained by catalysts based on the recommendations from exploration (PV acquisition function) and exploitation (EI acquisition function) in Phase 1. Reaction conditions: H_2:CO = 2.0, T = 533 K, P = 50 bar, and $GHSV$ = 24,000 cm^3 h^{-1} g_{cat}^{-1}.

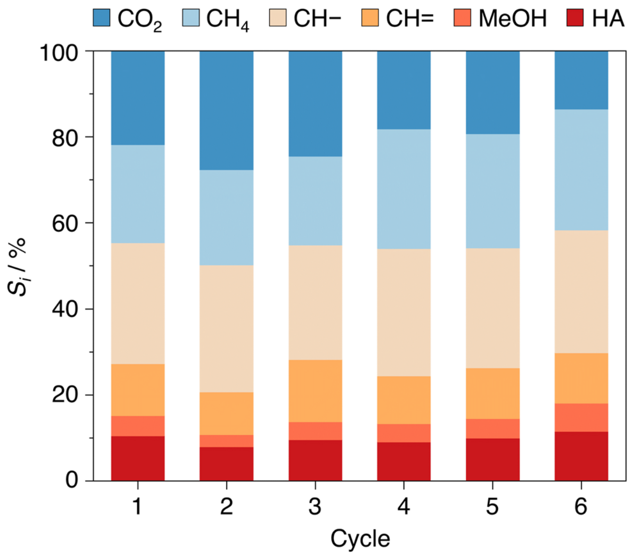

Supplementary Figure 8 | Product distribution for FeCoCuZr catalysts attaining the highest STY_{HA} in each active learning cycle of Phase 2. Catalyst compositions, reaction conditions, and full selectivity distribution of C_{2+} products are detailed in **Supplementary Tables 14-20**.

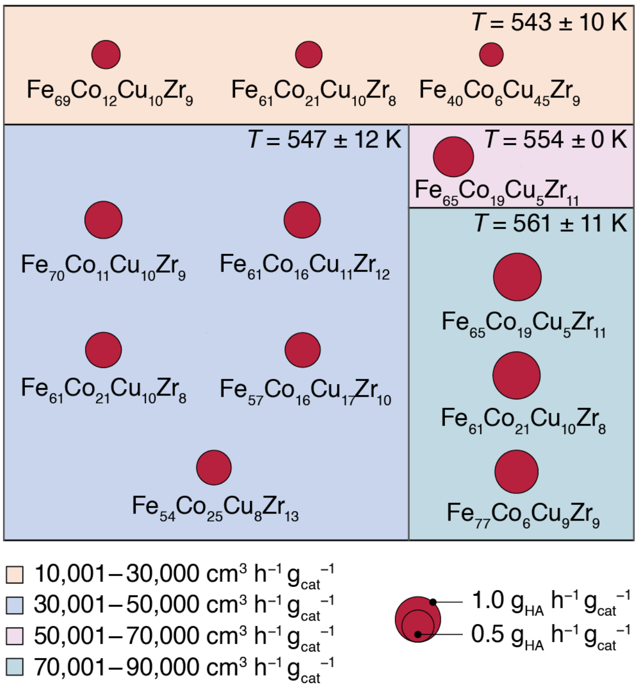

Supplementary Figure 9 | Impact of reaction conditions on STY_{HA} as determined in Phase 2. Clusters are based on *GHSV* values after SHAP analysis (**Fig 5c**). The area of the clusters is proportional to the number of experiments with *GHSV* in the specified range. The average reaction temperature is indicated and STY_{HA} is related to the area of each circle.

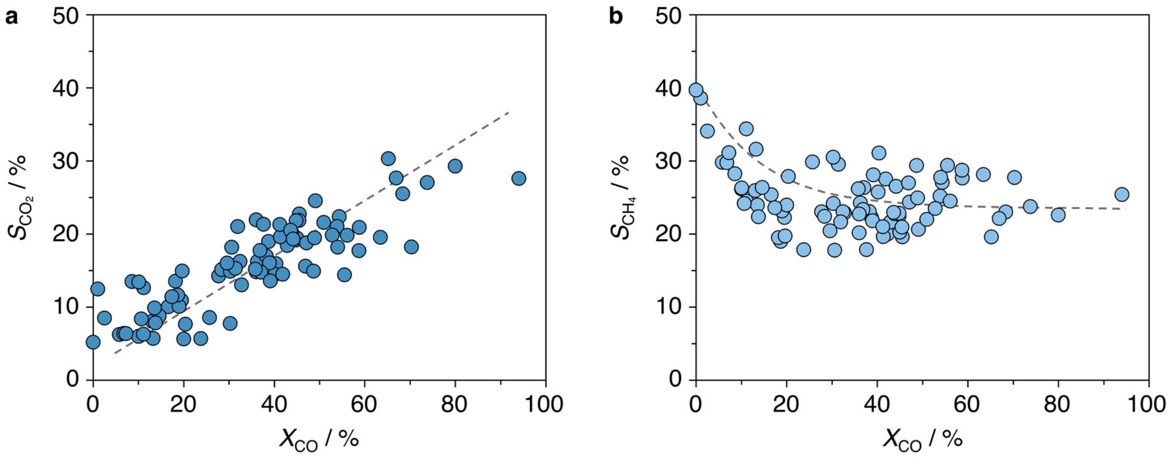

Supplementary Figure 10 I Correlation between selectivity toward **a**) CO_2 and **b**) CH_4 with CO conversion for all catalysts evaluated in Phase 1 and Phase 2.

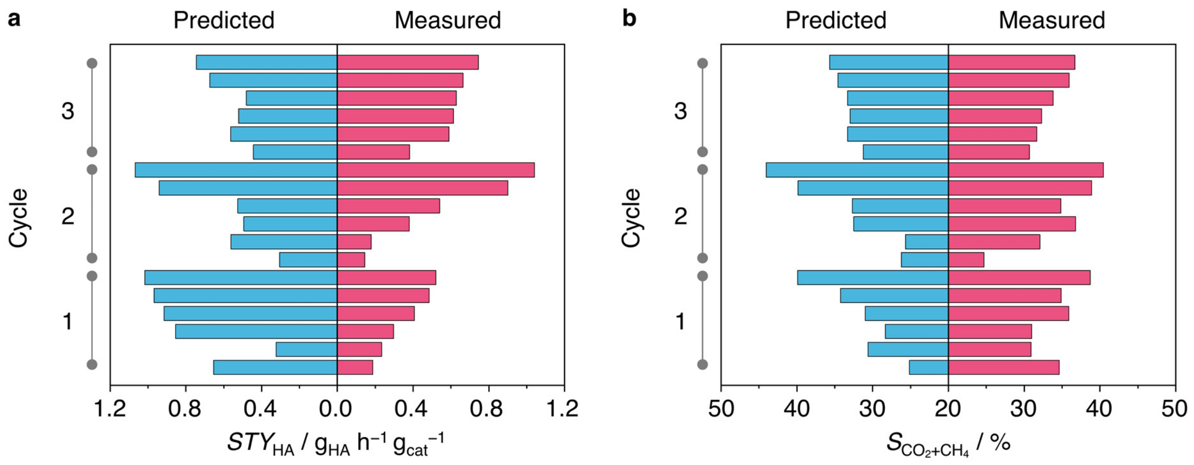

Supplementary Figure 11 I Model performance in terms of predicted and measured values of **a**) STY_{HA} and **b**) $S_{CO_2+CH_4}$ during Phase 3. The significant discrepancy between the predicted and measured values observed in the first cycle progressively improved by the second and third cycle.

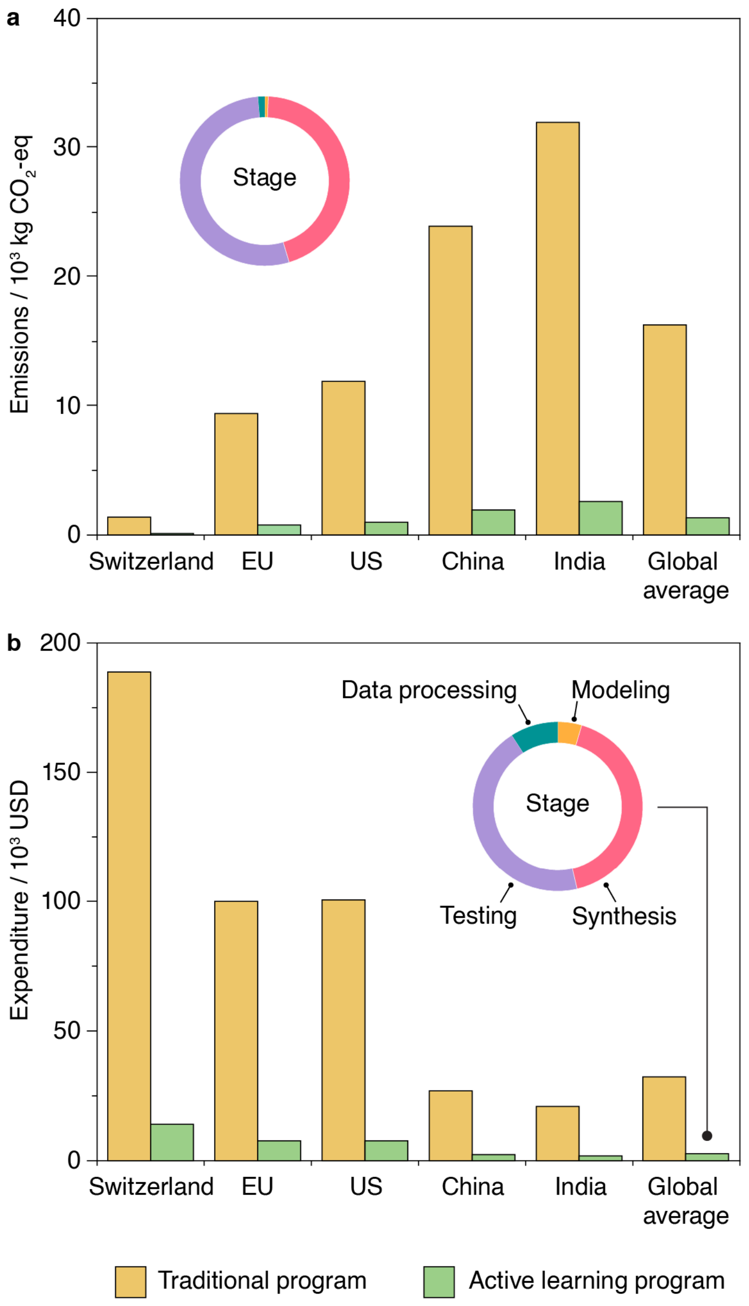

Supplementary Figure 12 | Comparison of **a**) environmental and **b**) economic costs between traditional and active learning programs for catalyst development according to **Supplementary Note 6**. The pie charts indicate the breakdown of costs for each stage (modeling, synthesis, testing, and data processing) in the case of the active learning program using global average values as a representative case. Numerical values are available in **Supplementary Tables 27-29**.

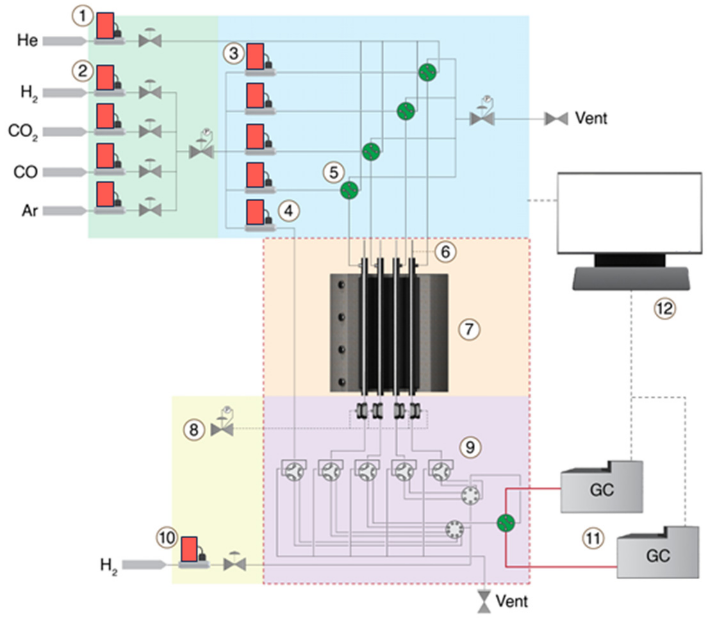

Supplementary Figure 13 | Simplified piping and instrumentation diagram of the high-pressure continuous-flow setup comprising four parallel fixed-bed reactors used in this study. Main parts of the setup are shaded with the same color in both panels. The setup features mass flow controllers (1) to purge reactors with He, (2) to prepare mixtures of reactant gases, and (3) to feed the mixtures into the reactors; (4) a dedicated line to analyze mixtures of reactants, (5) valves to switch between purge and mixture feed lines, (6) thermocouples, (7) reactors housed in the furnace, (8) back-pressure regulators and a control line, (9) valves to sample the reactor outlet streams, (10) carrier gas lines to carry stored samples from each sample loop to the respective gas chromatograph, (11) online gas chromatographs (Agilent 8890 equipped with Agilent PoraPLOT Q and Restek ShinCarbon columns), and (12) computer control using Process@ software from PID Eng&Tech. All mass flow controllers are supplied by Bronkhorst (El-Flow F-201CV). Solid lines indicate fluid-containing pipes and heated parts are outlined in red.

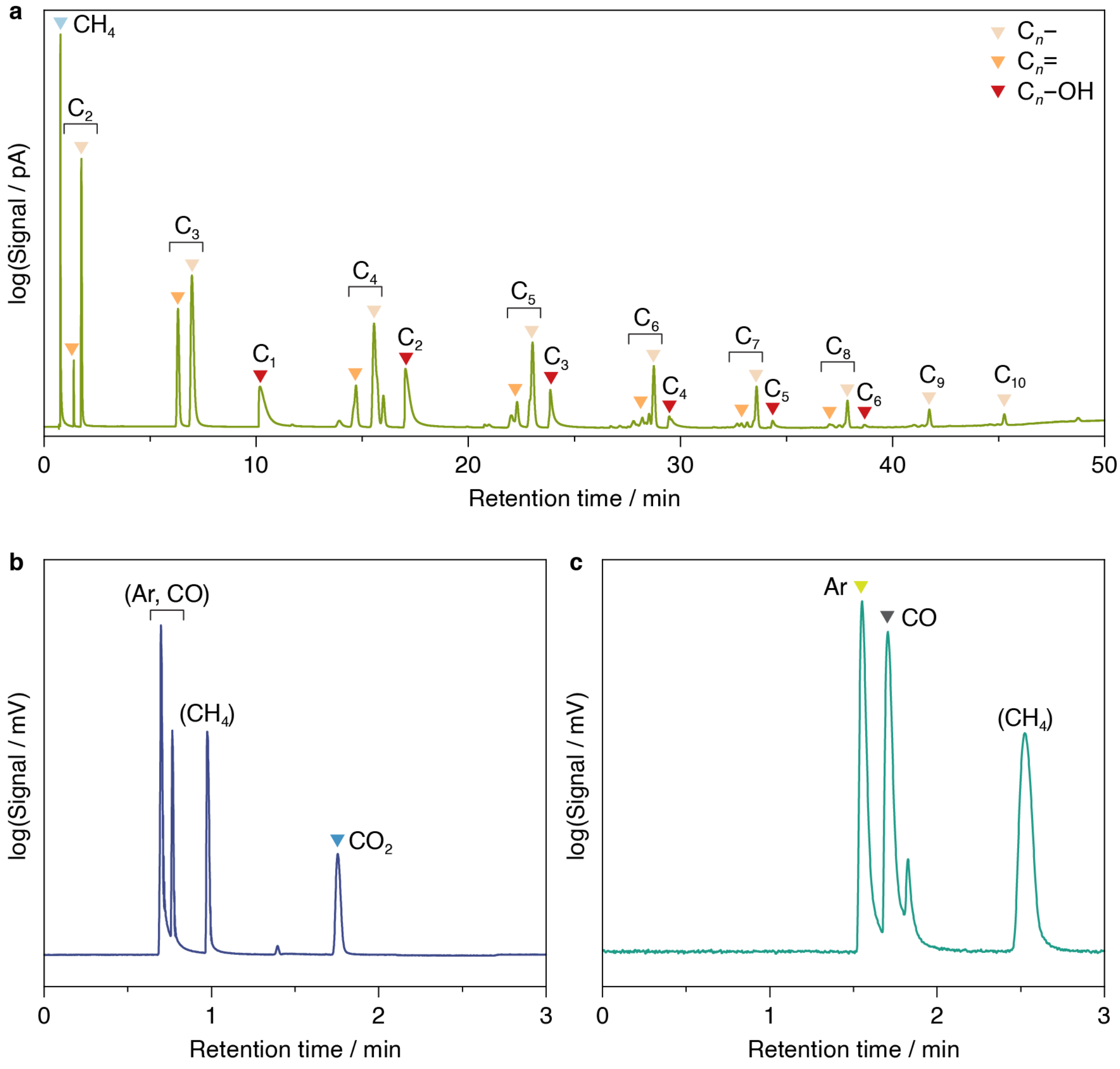

Supplementary Figure 14 | Representative chromatograms obtained from analysis of the effluent stream, as measured by the **a**) flame ionization detector, **b**) thermal conductivity detector, and by the **c**) auxiliary thermal conductivity detector post-separation of permanent gases. Case shown: $Fe_{69}Co_{12}Cu_{10}Zr_{9}$ in Cycle 3, Phase 1 (**Supplementary Table 5**). Peaks marked with colored triangles in the respective chromatograms were integrated and used in the quantification of their corresponding product. Parentheses indicate the detection of a compound by another detector other than the one used for quantification. Products in **a**) are classified based on carbon number and functional group (alkanes, alkenes, and alcohols).

Supplementary Tables

Supplementary Table 1 | Chemical composition and catalytic performance for seed catalysts in Phase 1. Reaction conditions: H_2:CO = 2.0, T = 533 K, P = 50 bar, and $GHSV$ = 24,000 cm^3 h^{-1} g$_{cat}$$^{-1}$.

Exp. #	Composition / mol %				X_{CO} / %	Selectivity / %						STY_{HA} / g_{HA} h^{-1} g_{cat}^{-1}
	Fe	Co	Cu	Zr		HA	MeOH	CH=	CH-	CH_4	CO_2	
1	87	0	0	13	47	7	3	28	26	14	22	0.24
2	68	19	0	14	30	14	7	16	28	23	13	0.29
3	57	32	0	12	35	12	5	19	29	20	15	0.28
4	42	46	0	12	36	11	3	29	28	17	14	0.24
5	27	61	0	13	68	4	1	30	38	17	11	0.16
6	16	71	0	13	70	2	1	10	47	25	15	0.10
7	0	0	89	11	0	0	55	2	1	36	6	0.00
8	17	0	70	13	10	9	10	12	28	26	15	0.09
9	29	0	60	11	18	9	7	13	33	22	15	0.13
10	45	0	43	12	34	8	5	9	37	16	24	0.21
11	54	0	33	12	48	9	5	12	35	15	24	0.30
12	72	0	16	12	55	8	4	14	35	15	24	0.31
13	0	85	0	15	93	0	0	2	49	29	20	0.00
14	0	70	18	12	11	9	4	10	33	42	1	0.09

(continued on next page)

(continued from previous page)

Exp. #	Composition / mol %				X_{CO} / %	Selectivity / %						STY_{HA} / g_{HA} h^{-1} g_{cat}^{-1}
	Fe	Co	Cu	Zr		HA	MeOH	CH=	CH-	CH_4	CO_2	
15	0	57	31	12	13	8	3	15	35	36	3	0.09
16	0	44	46	10	15	7	2	18	38	30	4	0.09
17	0	27	62	12	13	8	2	21	34	32	3	0.08
18	0	17	71	12	10	9	2	22	36	28	3	0.08
19	79	10	0	11	36	13	7	16	28	21	15	0.32
20	81	0	0	19	34	9	4	26	29	16	17	0.22
21	0	49	45	6	8	8	4	17	32	36	3	0.06
22	0	43	40	18	20	9	2	20	38	28	3	0.14
23	0	31	31	39	9	9	2	24	29	32	4	0.07
24	68	0	32	0	26	8	3	20	37	18	14	0.16
25	64	0	30	6	43	7	4	14	35	16	23	0.25
26	57	0	25	18	29	11	7	15	32	18	17	0.24
27	78	22	0	0	26	13	7	13	30	25	11	0.25
28	74	20	0	6	35	12	6	14	28	23	17	0.30
29	62	18	0	20	37	13	6	16	28	23	16	0.32
30	44	12	0	44	8	14	9	18	23	27	9	0.12
31	41	0	20	38	11	7	8	19	30	24	12	0.06

Supplementary Table 2 | Upper and lower compositional bounds applied across cycles in Phase 1.

Cycle #	Fe / mol%	Co / mol%	Cu / mol%	Zr / mol%
1	0-100	0-100	0-100	0-20
2	0-100	0-100	0-100	0-20
3	0-100	0-100	0-100	0-20
4	40-70	40-70	40-70	40-70
5	40-70	40-70	40-70	40-70

Supplementary Table 3 | Chemical composition and catalytic performance for catalysts in Cycle 1 of Phase 1. Reaction conditions: H_2:CO = 2.0, T = 533 K, P = 50 bar, and $GHSV$ = 24,000 cm^3 h^{-1} g$_{cat}^{-1}$.

Exp. #	Composition / mol %				X_{CO} / %	Selectivity / %						STY_{HA} / g_{HA} h^{-1} g_{cat}^{-1}
	Fe	Co	Cu	Zr		HA	MeOH	CH=	CH-	CH_4	CO_2	
1	21	21	48	10	13	14	8	20	24	26	8	0.16
2	22	48	20	10	24	11	2	31	33	18	6	0.22
3	36	10	42	12	17	16	9	13	27	25	10	0.24
4	47	23	20	10	20	15	6	18	27	22	11	0.27
5	36	40	11	13	19	16	5	19	26	23	10	0.28
6	61	16	11	13	28	15	8	14	27	23	14	0.30

Supplementary Table 4 | Chemical composition and catalytic performance for catalysts in Cycle 2 of Phase 1. Reaction conditions: H_2:CO = 2.0, T = 533 K, P = 50 bar, and $GHSV$ = 24,000 cm^3 h^{-1} g_{cat}^{-1}.

Exp. #	Composition / mol %				X_{CO} / %	Selectivity / %						STY_{HA} / g_{HA} h^{-1} g_{cat}^{-1}
	Fe	Co	Cu	Zr		HA	MeOH	CH=	CH-	CH_4	CO_2	
1	27	5	5	62	9	12	8	24	14	28	13	0.04
2	24	36	35	5	10	11	4	25	27	26	6	0.07
3	28	21	27	24	11	14	7	19	22	24	13	0.12
4	33	34	5	28	19	14	4	27	24	19	12	0.18
5	60	18	7	15	28	14	6	13	28	22	15	0.32
6	61	21	10	8	33	14	7	13	30	23	13	0.34

Supplementary Table 5 | Chemical composition and catalytic performance for catalysts in Cycle 3 of Phase 1. Reaction conditions: H_2:CO = 2.0, T = 533 K, P = 50 bar, and $GHSV$ = 24,000 $cm^3\ h^{-1}\ g_{cat}^{-1}$.

Exp. #	Composition / mol %				X_{CO} / %	Selectivity / %						STY_{HA} / $g_{HA}\ h^{-1}\ g_{cat}^{-1}$
	Fe	Co	Cu	Zr		HA	MeOH	CH=	CH-	CH_4	CO_2	
1	5	5	6	85	1	7	5	26	11	39	12	0.01
2	5	5	39	50	3	7	7	24	19	34	9	0.02
3	5	38	5	51	13	12	4	19	28	32	6	0.10
4	71	13	6	11	36	13	8	12	27	24	16	0.34
5	81	6	6	8	42	11	6	14	29	20	20	0.34
6	69	12	10	9	45	12	6	12	28	22	19	0.39

Supplementary Table 6 | Chemical composition and catalytic performance for catalysts in Cycle 4 of Phase 1. Reaction conditions: H_2:CO = 2.0, T = 533 K, P = 50 bar, and $GHSV$ = 24,000 $cm^3\ h^{-1}\ g_{cat}^{-1}$.

Exp. #	Composition / mol %				X_{CO} / %	Selectivity / %						STY_{HA} / $g_{HA}\ h^{-1}\ g_{cat}^{-1}$
	Fe	Co	Cu	Zr		HA	MeOH	CH=	CH-	CH_4	CO_2	
1	22	6	44	28	10	10	10	15	26	26	13	0.06
2	36	6	5	53	6	13	7	24	20	30	6	0.06
3	57	16	17	11	30	12	7	12	30	24	15	0.26
4	65	19	5	11	32	13	6	13	29	23	16	0.30
5	77	6	9	9	46	11	5	13	29	20	23	0.34
6	71	9	11	9	43	13	7	13	28	22	18	0.38

Supplementary Table 7 | Chemical composition and catalytic performance for catalysts in Cycle 5 of Phase 1. Reaction conditions: H_2:CO = 2.0, T = 533 K, P = 50 bar, and $GHSV$ = 24,000 $cm^3\ h^{-1}\ g_{cat}^{-1}$.

Exp. #	Composition / mol %				X_{CO} / %	Selectivity / %						STY_{HA} / $g_{HA}\ h^{-1}\ g_{cat}^{-1}$
	Fe	Co	Cu	Zr		HA	MeOH	CH=	CH-	CH_4	CO_2	
1	5	26	47	21	20	11	2	22	35	24	6	0.14
2	23	6	66	5	15	14	10	14	27	26	9	0.14
3	42	17	6	34	17	15	7	18	25	24	11	0.18
4	63	14	12	11	39	13	6	11	29	22	19	0.35
5	74	11	5	9	38	13	7	12	27	23	17	0.36
6	76	6	13	7	45	12	6	13	30	20	20	0.38

Supplementary Table 8 | Selectivity distribution of C_{2+} products for catalysts attaining the highest STY_{HA} in each cycle of Phase 1 as detailed in **Supplementary Tables 3-7**.

	Length	Cycle 1	Cycle 2	Cycle 3	Cycle 4	Cycle 5
Alkane (CH-) selectivity / %	C_2	10.6	12.2	11.1	10.7	10.5
	C_3	5.5	6.9	6.2	6.1	6.4
	C_4	4.1	4.2	4.0	4.1	4.5
	C_5	2.4	2.5	2.5	2.6	2.9
	C_6	1.5	1.6	1.5	1.6	1.9
	C_7	1.0	1.1	1.1	1.2	1.4
	C_{8+}	1.3	1.6	1.5	1.6	2.0
Alkene (CH=) selectivity / %	C_2	1.0	0.9	0.8	0.9	0.8
	C_3	7.0	6.8	6.1	6.2	6.1
	C_4	3.0	2.9	2.6	2.7	2.8
	C_5	1.8	1.6	1.5	1.7	1.8
	C_6	0.6	0.6	0.5	0.6	0.7
	C_7	0.2	0.2	0.2	0.2	0.3
	C_{8+}	0.0	0.1	0.1	0.1	0.1
Higher alcohol (HA) selectivity / %	C_2	8.8	8.2	7.9	7.7	7.1
	C_3	4.1	4.1	3.3	3.3	3.2
	C_4	1.4	1.3	1.0	1.1	0.9
	C_{5+}	0.5	0.7	0.1	0.6	0.7

Supplementary Table 9 | Structural properties of $Fe_{69}Co_{12}Cu_{10}Zr_9$ at various stages of the reaction. Reaction conditions: H_2:CO = 2.0, T = 533 K, P = 50 bar, and $GHSV$ = 24,000 $cm^3\ h^{-1}\ g_{cat}^{-1}$.

Status	TOS / h	d_{Fe3O4} / nm	S_{BET} / $m^2\ g_{cat}^{-1}$
Fresh	-	9	136
Reduced	0	9	52
Used	24	15	24
Used	100	15	20

Supplementary Table 10 | Rate of alcohol formation per unit area of $Fe_{69}Co_{12}Cu_{10}Zr_9$ and $Fe_{79}Co_{10}Zr_{11}$ catalysts. Reaction conditions: H_2:CO = 2.0, T = 533 K, P = 50 bar, and $GHSV$ = 24,000 cm^3 h^{-1} g_{cat}^{-1}.

**Catalyst**	**S_{BET} / m^2 g_{cat}^{-1}**		**Rate of alcohol formation / mmol h^{-1} m_{cat}^{-2}**			
	**Fresh**	**Used**	**C_1**	**C_2**	**C_3**	**C_{4+}**
$Fe_{79}Co_{10}Zr_{11}$	146	38	0.20	0.12	0.03	0.01
$Fe_{69}Co_{12}Cu_{10}Zr_9$	136	24	0.39	0.24	0.07	0.02

Supplementary Table 11 | Predicted and measured performance metrics for catalysts in Phase 1.

Cycle #	Exp. #	Acquisition function	STY_{HA} / g_{HA} h^{-1} g_{cat}^{-1}		
			Predicted	Measured	Error
1	1	PV	0.14	0.16	+0.02
	2	PV	0.16	0.22	+0.06
	3	PV	0.20	0.24	+0.04
	4	PV	0.27	0.27	+0.00
	5	PV	0.23	0.28	+0.05
	6	EI	0.32	0.30	–0.02
2	1	PV	0.13	0.04	–0.09
	2	PV	0.17	0.07	–0.10
	3	PV	0.22	0.12	–0.10
	4	PV	0.23	0.18	–0.05
	5	EI	0.32	0.32	+0.00
	6	EI	0.30	0.34	+0.04
3	1	PV	0.16	0.01	–0.15
	2	PV	0.09	0.02	–0.07
	3	PV	0.11	0.10	–0.01
	4	EI	0.32	0.34	+0.02
	5	EI	0.31	0.34	+0.03
	6	EI	0.32	0.39	+0.07
4	1	PV	0.05	0.06	+0.01
	2	PV	0.08	0.06	–0.02
	3	EI	0.34	0.26	–0.08
	4	EI	0.33	0.30	–0.03
	5	EI	0.36	0.34	–0.02
	6	EI	0.36	0.38	+0.02
5	1	PV	0.11	0.14	+0.03
	2	PV	0.06	0.14	+0.08
	3	PV	0.13	0.18	+0.05
	4	EI	0.32	0.35	+0.03
	5	EI	0.33	0.36	+0.03
	6	EI	0.34	0.38	+0.04

Supplementary Table 12 | Chemical composition, reaction conditions, and catalytic performance for seed catalysts in Phase 2.

Exp. #	Composition / mol %				*T* / K	*P* / bar	*GHSV* / $cm^3\ h^{-1}\ g_{cat}^{-1}$	H_2:CO / -	X_{CO} / %	Selectivity / %						STY_{HA} / $g_{HA}\ h^{-1}\ g_{cat}^{-1}$
	Fe	Co	Cu	Zr						HA	MeOH	CH=	CH-	CH_4	CO_2	
1	21	21	48	10	523	50	30,000	1.5	7	11	6	25	21	30	6	0.07
2	21	21	48	10	543	50	27,000	1.5	14	11	5	23	26	24	10	0.15
3	21	21	48	10	513	50	42,000	3.0	0	10	11	16	18	40	5	0.05
4	21	21	48	10	533	50	36,000	3.0	11	12	10	13	25	34	6	0.13
5	21	21	48	10	553	50	24,000	2.5	31	10	6	10	30	30	15	0.19
6	36	40	11	13	523	50	12,000	1.5	18	14	4	24	26	20	14	0.09
7	36	40	11	13	533	50	18,000	1.5	20	12	3	23	27	20	15	0.16
8	36	40	11	13	513	50	24,000	3.0	7	14	8	16	24	31	6	0.09
9	36	40	11	13	543	50	27,000	3.0	40	10	5	9	29	31	16	0.25
10	36	40	11	13	553	50	24,000	2.5	54	8	3	9	31	27	22	0.28
11	61	21	10	8	513	50	27,000	1.5	14	14	7	21	27	22	8	0.13
12	61	21	10	8	543	50	30,000	1.5	45	10	4	15	28	21	22	0.48
13	61	21	10	8	523	50	36,000	3.0	30	15	11	8	28	31	8	0.39
14	61	21	10	8	533	50	18,000	3.0	70	11	6	6	32	28	18	0.32
15	61	21	10	8	553	50	24,000	2.5	94	6	2	5	34	25	28	0.34
16	69	12	10	9	513	50	36,000	1.5	11	15	10	18	25	24	8	0.16
17	69	12	10	9	543	50	27,000	1.5	36	11	5	15	27	20	22	0.39
18	69	12	10	9	523	50	18,000	3.0	40	14	9	8	29	26	14	0.23
19	69	12	10	9	533	50	30,000	3.0	42	13	9	8	28	28	15	0.37
20	69	12	10	9	553	50	24,000	2.5	80	7	4	6	31	23	29	0.39

Supplementary Table 13 | Upper and lower compositional and operational bounds applied across cycles in Phase 2.

Cycle #	Fe / mol %	Co / mol %	Cu / mol %	Zr / mol %	H_2:CO / -	*T* / K	*P* / bar	*GHSV* / $cm^3\ h^{-1}\ g_{cat}^{-1}$
1	40-80	5-25	5-25	10	1.0-3.0	523-593	50	10,000-50,000
2	40-80	5-25	5-25	10	1.0-3.0	523-593	50	10,000-50,000
3	40-80	5-25	5-25	10	1.0-3.0	523-593	50	10,000-50,000
4	40-80	5-25	5-25	10	1.0-3.0	523-593	50	10,000-90,000
5	40-80	5-25	5-25	10	1.0-3.0	523-593	50	10,000-90,000
6	40-80	5-25	5-25	10	1.0-3.0	523-593	50	10,000-90,000

Supplementary Table 14 | Chemical composition, reaction conditions, and catalytic performance for catalysts in Cycle 1 of Phase 2.

Exp. #	Composition / mol %				*T* / K	*P* / bar	*GHSV* / $cm^3 h^{-1} g_{cat}^{-1}$	H_2:CO / -	X_{CO} / %	Selectivity / %						STY_{HA} / $g_{HA} h^{-1} g_{cat}^{-1}$
	Fe	Co	Cu	Zr						HA	MeOH	CH=	CH-	CH_4	CO_2	
1	43	38	9	10	538	50	10,250	2.0	41	11	3	13	33	20	20	0.14
2	61	20	9	10	519	50	33,000	3.2	20	16	12	10	26	28	8	0.25
3	40	6	45	9	547	50	20,000	2.0	44	11	7	8	31	23	20	0.26
4	61	20	9	10	552	50	19,000	1.8	65	8	3	9	30	20	3	0.34
5	61	20	9	10	530	50	43,500	3.2	26	14	11	9	28	30	9	0.39
6	61	20	9	10	552	50	32,550	1.8	45	10	5	12	28	23	22	0.54

Supplementary Table 15 | Chemical composition, reaction conditions, and catalytic performance for catalysts in Cycle 2 of Phase 2.

Exp. #	Composition / mol %				*T* / K	*P* / bar	*GHSV* / $cm^3 h^{-1} g_{cat}^{-1}$	H_2:CO / -	X_{CO} / %	Selectivity / %						STY_{HA} / $g_{HA} h^{-1} g_{cat}^{-1}$
	Fe	Co	Cu	Zr						HA	MeOH	CH=	CH-	CH_4	CO_2	
1	70	11	10	9	546	50	32,600	2.7	68	8	4	6	33	23	26	0.43
2	64	14	10	12	550	50	37,500	2.4	54	10	5	9	28	25	21	0.53
3	54	25	8	13	554	50	43,800	1.8	39	12	4	16	30	22	16	0.57
4	54	25	8	13	554	50	47,200	1.4	30	12	4	20	27	20	16	0.58
5	63	14	12	11	557	50	46,300	1.2	32	10	4	17	26	22	21	0.60
6	61	21	10	8	552	50	40,700	2.0	67	8	3	10	30	22	28	0.63

Supplementary Table 16 | Chemical composition, reaction conditions, and catalytic performance for catalysts in Cycle 3 of Phase 2.

Exp. #	Composition / mol %				*T* / K	*P* / bar	*GHSV* / $cm^3 h^{-1} g_{cat}^{-1}$	H_2:CO / -	X_{CO} / %	Selectivity / %						STY_{HA} / $g_{HA} h^{-1} g_{cat}^{-1}$
	Fe	Co	Cu	Zr						HA	MeOH	CH=	CH-	CH_4	CO_2	
1	50	28	12	10	546	50	37,460	1.3	31	12	4	21	27	18	18	0.43
2	50	28	12	10	551	50	36,910	1.4	38	11	3	19	28	18	21	0.49
3	61	20	9	10	550	50	38,060	1.4	41	11	4	16	26	21	21	0.53
4	57	16	17	11	547	50	37,590	2.0	47	12	6	11	28	24	19	0.60
5	61	16	11	13	553	50	48,240	1.7	37	12	6	14	26	23	18	0.64
6	70	11	10	9	554	50	49,440	1.7	49	10	4	15	27	21	25	0.67

Supplementary Table 17 | Chemical composition, reaction conditions, and catalytic performance for catalysts in Cycle 4 of Phase 2.

Exp. #	Composition / mol %				*T* / K	*P* / bar	*GHSV* / $cm^3\ h^{-1}\ g_{cat}^{-1}$	H_2:CO / -	X_{CO} / %	Selectivity / %						STY_{HA} / $g_{HA}\ h^{-1}\ g_{cat}^{-1}$
	Fe	Co	Cu	Zr						HA	MeOH	CH=	CH-	CH_4	CO_2	
1	58	19	15	7	554	50	39,710	2.2	53	10	4	11	32	24	20	0.52
2	66	5	22	7	555	50	50,000	2.2	51	9	5	12	30	22	22	0.58
3	71	13	6	11	555	50	55,200	1.8	44	11	5	13	28	23	21	0.76
4	65	19	5	11	555	50	58,750	2.2	49	11	5	12	29	25	19	0.78
5	77	6	9	9	544	50	80,000	2.0	36	12	7	15	28	23	15	0.88
6	61	21	10	8	562	50	76,500	2.5	54	9	4	11	30	28	18	0.92

Supplementary Table 18 | Chemical composition, reaction conditions, and catalytic performance for catalysts in Cycle 5 of Phase 2.

Exp. #	Composition / mol %				*T* / K	*P* / bar	*GHSV* / $cm^3\ h^{-1}\ g_{cat}^{-1}$	H_2:CO / -	X_{CO} / %	Selectivity / %						STY_{HA} / $g_{HA}\ h^{-1}\ g_{cat}^{-1}$
	Fe	Co	Cu	Zr						HA	MeOH	CH=	CH-	CH_4	CO_2	
1	50	28	12	10	587	50	80,000	2.4	74	5	2	10	33	24	27	0.68
2	71	13	6	11	561	50	80,000	3.0	63	8	5	8	31	28	20	0.86
3	65	19	5	11	559	50	72,300	2.7	59	9	4	9	30	28	21	0.89
4	77	6	9	9	555	50	80,000	2.9	56	9	6	11	30	25	20	0.90
5	61	21	10	8	560	50	90,000	2.8	56	10	5	9	32	29	14	1.09
6	65	19	5	11	560	50	90,000	2.2	44	10	5	12	28	27	19	1.10

Supplementary Table 19 | Chemical composition, reaction conditions, and catalytic performance for catalysts in Cycle 6 of Phase 2.

Exp. #	Composition / mol %				*T* / K	*P* / bar	*GHSV* / $cm^3\ h^{-1}\ g_{cat}^{-1}$	H_2:CO / -	X_{CO} / %	Selectivity / %						STY_{HA} / $g_{HA}\ h^{-1}\ g_{cat}^{-1}$
	Fe	Co	Cu	Zr						HA	MeOH	CH=	CH-	CH_4	CO_2	
1	66	5	23	7	557	50	80,000	3.0	47	10	7	10	31	27	16	0.78
2	65	19	5	11	557	50	74,320	3.0	59	9	5	8	31	29	18	0.86
3	64	14	10	12	557	50	80,000	3.0	49	10	6	9	30	29	15	0.90
4	60	18	7	15	556	50	80,000	2.1	37	12	6	13	28	26	15	0.93
5	60	18	7	15	557	50	80,000	2.0	36	12	6	13	28	26	15	0.94
6	64	14	10	12	557	50	90,000	2.5	39	11	7	12	29	28	14	1.03

Supplementary Table 20 | Selectivity distribution of C_{2+} products for catalysts attaining the highest STY_{HA} in each cycle of Phase 2 as detailed in **Supplementary Tables 14-19.

	Length	Cycle 1	Cycle 2	Cycle 3	Cycle 4	Cycle 5	Cycle 6
Alkane (CH-) selectivity / %	C_2	11.3	11.7	10.0	13.0	12.0	11.7
	C_3	6.3	7.2	5.5	7.2	6.5	6.1
	C_4	3.8	4.2	3.5	3.6	3.8	4.2
	C_5	2.5	2.5	2.7	2.4	2.2	2.5
	C_6	1.5	1.4	1.7	1.3	1.3	1.4
	C_7	1.0	1.0	1.2	0.8	0.9	1.0
	C_{8+}	1.8	1.5	2.1	1.4	1.3	1.6
Alkene (CH=) selectivity / %	C_2	0.9	0.6	1.1	0.8	0.9	0.9
	C_3	6.4	5.2	6.9	5.8	5.9	6.1
	C_4	2.7	2.1	3.2	2.3	2.5	2.5
	C_5	1.6	1.2	2.1	1.4	1.5	1.5
	C_{6+}	0.2	0.5	0.7	0.5	0.6	0.5
	C_7	0.2	0.2	0.3	0.2	0.2	0.2
	C_{8+}	0.1	0.1	0.2	0.1	0.1	0.1
Higher alcohol (HA) selectivity / %	C_2	6.1	4.7	5.6	5.7	6.3	7.5
	C_3	2.8	2.1	2.5	2.3	2.5	2.8
	C_4	1.0	0.7	0.9	0.7	0.8	0.8
	C_{5+}	0.5	0.4	0.5	0.3	0.4	0.4

Supplementary Table 21 | Predicted and measured performance metrics for catalysts in Phase 2.

Cycle #	Exp. #	Acquisition function	STY_{HA} / g_{HA} h^{-1} g_{cat}^{-1}			Cycle #	Exp. #	Acquisition function	STY_{HA} / g_{HA} h^{-1} g_{cat}^{-1}		
			Predicted	Measured	Error				Predicted	Measured	Error
1	1	PV	0.28	0.14	–0.14	4	1	PV	0.56	0.52	–0.04
	2	PV	0.33	0.25	–0.08		2	EI	0.64	0.58	–0.06
	3	PV	0.32	0.26	–0.06		3	EI	0.68	0.76	+0.08
	4	EI	0.45	0.34	–0.11		4	EI	0.66	0.78	+0.12
	5	EI	0.41	0.39	–0.02		5	EI	0.55	0.88	+0.33
	6	EI	0.49	0.54	+0.05		6	EI	0.54	0.92	+0.38
2	1	PV	0.46	0.43	–0.03	5	1	EI	0.74	0.68	–0.06
	2	PV	0.63	0.53	+0.02		2	EI	0.86	0.86	+0.00
	3	EI	0.56	0.57	+0.01		3	EI	0.85	0.89	+0.04
	4	EI	0.56	0.58	+0.02		4	EI	0.85	0.90	+0.05
	5	EI	0.55	0.60	+0.05		5	EI	0.94	1.09	+0.15
	6	EI	0.54	0.63	+0.09		6	EI	0.98	1.10	+0.12
3	1	PV	0.58	0.43	–0.15	6	1	PV	0.92	0.78	–0.15
	2	PV	0.59	0.49	–0.10		2	EI	0.89	0.86	–0.03
	3	EI	0.63	0.53	–0.10		3	EI	0.95	0.90	–0.05
	4	EI	0.55	0.60	+0.05		4	EI	1.00	0.93	–0.07
	5	EI	0.63	0.64	+0.01		5	EI	1.00	0.94	–0.06
	6	EI	0.62	0.67	+0.05		6	EI	1.07	1.03	–0.04

Supplementary Table 22 | Chemical composition, reaction conditions, and catalytic performance for catalysts in Cycle 1 of Phase 3.

Exp. #	Composition / mol %				*T* / K	*P* / bar	*GHSV* / $cm^3\ h^{-1}\ g_{cat}^{-1}$	H_2:CO / -	X_{CO} / %	Selectivity / %						STY_{HA} / $g_{HA}\ h^{-1}\ g_{cat}^{-1}$
	Fe	Co	Cu	Zr						HA	MeOH	CH=	CH-	CH_4	CO_2	
1	41	35	17	8	530	50	77,400	1.9	10	13	7	27	18	31	4	0.19
2	81	6	6	8	507	50	80,000	2.8	11	15	15	14	24	25	6	0.23
3	34	45	14	7	543	50	80,000	2.0	15	11	4	26	28	26	5	0.30
4	43	38	9	10	545	50	80,000	2.1	20	12	6	21	25	27	9	0.41
5	50	28	12	10	547	50	80,000	2.2	21	11	5	21	28	24	11	0.48
6	54	25	8	13	552	50	80,000	2.2	24	11	5	18	26	27	11	0.52

Supplementary Table 23 | Chemical composition, reaction conditions, and catalytic performance for catalysts in Cycle 2 of Phase 3.

Exp. #	Composition / mol %				*T* / K	*P* / bar	*GHSV* / $cm^3\ h^{-1}\ g_{cat}^{-1}$	H_2:CO / -	X_{CO} / %	Selectivity / %						STY_{HA} / $g_{HA}\ h^{-1}\ g_{cat}^{-1}$
	Fe	Co	Cu	Zr						HA	MeOH	CH=	CH-	CH_4	CO_2	
1	29	49	11	11	530	50	41,760	1.6	15	12	2	34	28	22	3	0.14
2	41	43	9	7	525	50	75,000	1.6	11	13	4	31	19	29	3	0.18
3	55	18	19	8	541	50	80,000	1.9	20	12	6	16	29	27	10	0.38
4	71	9	11	9	531	50	80,000	2.3	21	15	12	13	25	26	8	0.54
5	75	5	11	8	550	50	80,000	1.7	35	12	6	16	28	21	18	0.90
6	81	6	6	8	557	50	80,000	1.7	44	10	4	19	27	19	21	1.04

Supplementary Table 24 | Chemical composition, reaction conditions, and catalytic performance for catalysts in Cycle 3 of Phase 3.

Exp. #	Composition / mol %				*T* / K	*P* / bar	*GHSV* / cm^3 h^{-1} g_{cat}^{-1}	H_2:CO / -	X_{CO} / %	Selectivity / %						STY_{HA} / g_{HA} h^{-1} g_{cat}^{-1}
	Fe	Co	Cu	Zr						HA	MeOH	CH=	CH-	CH_4	CO_2	
1	50	28	12	10	542	50	56,820	1.5	21	13	5	24	28	19	11	0.38
2	81	6	6	8	529	50	80,000	1.9	20	15	9	19	26	24	8	0.59
3	81	6	6	8	529	50	80,000	2.1	23	15	9	17	27	25	8	0.61
4	81	6	6	8	529	50	80,000	3.0	26	15	10	14	27	26	8	0.63
5	71	9	11	9	538	50	80,000	2.0	24	14	10	15	25	25	11	0.66
6	71	9	11	9	541	50	80,000	1.9	25	14	9	15	25	25	12	0.75

Supplementary Table 25 | Selectivity distribution of C_{2+} products for catalysts in Cycle 3 of Phase 3 as detailed in **Supplementary Table 24**.

	Length	Exp. 1	Exp. 2	Exp. 3	Exp. 4	Exp. 5	Exp. 6
Alkane (CH-) selectivity / %	C_2	10.8	9.3	9.5	9.9	10.6	10.4
	C_3	5.4	4.5	4.9	5.3	5.3	5.3
	C_4	3.7	4.0	4.2	4.3	3.6	3.6
	C_5	2.4	2.6	2.8	2.7	2.2	2.2
	C_6	1.7	1.9	1.9	1.8	1.4	1.4
	C_7	1.4	1.4	1.4	1.2	1.0	1.0
	C_{8+}	2.2	1.9	2.0	1.7	1.4	1.4
Alkene (CH=) selectivity / %	C_2	2.3	2.4	1.6	1.2	1.5	1.4
	C_3	10.8	7.8	7.4	6.6	6.9	7.1
	C_4	5.5	4.1	3.8	3.1	3.2	3.3
	C_5	3.2	2.9	2.6	2.1	1.9	2.0
	C_{6+}	1.3	1.2	1.0	0.8	0.7	0.7
	C_7	0.6	0.6	0.4	0.3	0.3	0.3
	C_{8+}	0.3	0.3	0.3	0.2	0.1	0.2
Higher alcohol (HA) selectivity / %	C_2	6.4	8.9	9.1	9.3	8.9	8.8
	C_3	4.0	3.7	3.5	3.5	3.4	3.4
	C_4	1.6	1.3	1.3	1.2	1.2	1.2
	C_{5+}	1.0	0.9	0.9	0.8	0.7	0.7

Supplementary Table 26 | Predicted and measured performance metrics for catalysts in Phase 3.

Cycle #	Exp. #	Acquisition function	STY_{HA} / g_{HA} h^{-1} g_{cat}^{-1}			$S_{CO_2+CH_4}$ / %		
			Predicted	Measured	Error	Predicted	Measured	Error
1	1	EHVI	0.65	0.19	–0.46	25	35	+10
	2	EHVI	0.32	0.23	–0.09	31	31	+0
	3	EHVI	0.86	0.30	–0.56	28	32	+4
	4	EHVI	0.92	0.41	–0.51	31	36	+5
	5	EHVI	0.97	0.48	–0.49	34	35	+1
	6	EHVI	1.02	0.52	–0.50	40	39	–1
2	1	EHVI	0.30	0.15	–0.15	26	25	–1
	2	EHVI	0.56	0.18	–0.38	26	32	+6
	3	EHVI	0.49	0.38	–0.11	32	37	+5
	4	EHVI	0.53	0.54	+0.01	33	35	+2
	5	EHVI	0.94	0.90	–0.04	40	39	–1
	6	EHVI	1.07	1.04	–0.03	44	40	–4
3	1	EHVI	0.44	0.38	–0.06	31	31	+0
	2	EHVI	0.56	0.59	+0.03	33	32	–1
	3	EHVI	0.52	0.61	+0.09	33	32	–1
	4	EHVI	0.48	0.63	+0.15	33	34	+1
	5	EHVI	0.67	0.66	–0.01	35	36	+1
	6	EHVI	0.74	0.74	+0.00	36	37	+1

Supplementary Table 27 | Breakdown and assumptions in the estimation of environmental and economic savings.

Stage	Resource	Average quantity per catalyst	Unit
**Modeling**	Electricity (computer)	0.125	kWh
	Personnel	0.25	h
**Synthesis**	Electricity (ovens)	8	kWh
	Iron nitrate	0.007	kg
	Cobalt nitrate	0.001	kg
	Copper nitrate	0.001	kg
	Zr oxynitrate	0.001	kg
	Ethanol	0.2	kg
	Tartaric acid	0.008	kg
	Personnel	1	h
**Testing**	Electricity (setup)	10	kWh
	Hydrogen	0.0018	kg
	Carbon monoxide	0.0033	kg
	Personnel	2	h
**Data processing**	Electricity (computer)	0.25	kWh
	Personnel	0.5	h

Supplementary Table 28 | Environmental and economic costs of individual resources.

Resource	Location	Emissions / 10^3 kg CO_2-eq unit^{-1} [a][b]	Expenditure / 10^3 USD unit^{-1} [b]
**Electricity**	Switzerland	0.051	0.259[d]
	EU average	0.392	0.289[d]
	United States	0.496	0.147[d]
	China	1.003	0.090[d]
	India	1.342	0.131[d]
	Global average	0.681	0.150[d]
**Iron nitrate**	Global average	2.5[c]	168[e]
**Cobalt nitrate**	Global average	20.5[c]	708[e]
**Copper nitrate**	Global average	3.2[c]	141[e]
**Zr oxynitrate**	Global average	47[c]	528[e]
**Ethanol**	Global average	0.8[c]	8[e]
**Tartaric acid**	Global average	4.6[c]	113[e]
**Hydrogen**	Switzerland	10.132	337[e]
	EU average	9.845	
	United States	10.095	
	China	9.516	
	India	9.030	
	Global average	9.841	

(continued on next page)

(continued from previous page)

Resource	Location	Emissions / 10^3 kg CO_2-eq unit^{-1} [a][b]	Expenditure / 10^3 USD unit^{-1} [b]
**Carbon monoxide**	Switzerland	1.193	44[e]
	EU average		
	United States	2.023	
	China		
	India		
	Global average		
**Personnel[e]**	Switzerland	-	33.2[f]
	EU average		16.6[f]
	United States		17.3[f]
	China		3.9[f]
	India		2.6[f]
	Global average		4.7[f]

[a] Obtained from premise v1.5.8[13] based on Ecoinvent v3.8[14]. The inventories in premise were derived from the results obtained using the IMAGE Integrated Assessment Model[15] based on SSP2 for the year 2020.

[b] Units correspond to those listed in **Supplementary Table 22**.

[c] Estimated using the impact of the corresponding metal.

[d] June 2023 prices for business users.

[e] Based on actual purchase costs in March 2023 at ETH Zurich, Switzerland.

[f] Based on average PhD salaries reported.[17]

Supplementary Table 29 | Environmental and economic cost estimates of traditional and active learning experimental programs.

Program	Location	Emissions / 10^3 kg CO_2-eq					Expenditure / 10^3 USD				
		Modeling	Synthesis	Testing	Data processing	Total	Modeling	Synthesis	Testing	Data processing	Total
Traditional	Switzerland	-	0.28	1.07	0.03	1.38	-	16.1	139.4	33.3	188.8
	EU average	-	1.37	7.87	0.20	9.44	-	9.6	73.6	16.7	99.9
	United States	-	1.70	9.97	0.25	11.92	-	9.4	73.7	17.4	100.4
	China	-	3.32	20.11	0.50	23.94	-	3.9	19.0	4.0	26.9
	India	-	4.41	26.88	0.67	31.95	-	3.5	14.7	2.7	20.9
	Global average	-	2.29	13.67	0.34	16.30	-	4.4	23.2	4.8	32.3
Active learning	Switzerland	0.00	0.07	0.06	0.00	0.13	0.9	4.2	7.2	1.7	14.0
	EU average	0.01	0.36	0.41	0.01	0.78	0.4	2.5	3.8	0.9	7.6
	United States	0.01	0.44	0.52	0.01	0.98	0.5	2.4	3.8	0.9	7.6
	China	0.01	0.86	1.05	0.03	1.95	0.1	1.0	1.0	0.2	2.3
	India	0.02	1.15	1.40	0.03	2.60	0.1	0.9	0.8	0.1	1.9
	Global average	0.01	0.60	0.71	0.02	1.33	0.1	1.1	1.2	0.2	2.7

Supplementary Table 30 | Optimal model hyperparameters across the active learning phases.

Phase #	Cycle #	Lengthscale values / -						
		**Zr**	**Cu**	**Co**	**Fe**	***T***	**H_2:CO**	***GHSV***
1	1	0.16	0.56	7.75	0.47	N.A.	N.A.	N.A.
	2	0.15	35	0.43	0.53	N.A.	N.A.	N.A.
	3	0.14	0.62	0.63	0.64	N.A.	N.A.	N.A.
	4	0.15	0.36	0.46	972	N.A.	N.A.	N.A.
	5	0.16	0.41	0.49	7.93	N.A.	N.A.	N.A.
2	1	1.22	3060	1.29	0.42	0.81	2.36	1.52
	2	1.18	4130	1.40	0.41	0.85	2.25	1.09
	3	1.25	4370	1.47	0.39	0.80	1.76	1.08
	4	1.14	1670	1.35	0.39	0.71	1.01	0.99
	5	1.55	3480	2.00	0.42	0.77	2.00	0.89
	6	1.61	2200	2.11	0.42	0.52	2.25	0.91
3	1	0.69	639	0.72	0.43	0.39	0.80	0.77
	2	0.68	800	0.70	0.34	0.33	0.86	0.59
	3	0.68	738	0.64	0.31	0.34	0.84	0.55

Supplementary Table 31 | Stratified cross-validation results across the active learning phases.

Phase #	Metric	Full model	Stratified cross-validation
1	R^2	0.93	0.61
	MAPE	21.28%	49.21%
2	R^2	0.96	0.94
	MAPE	20.33%	7.85%
3	R^2	0.96	0.98
	MAPE	19.26%	17.03%

Supplementary References

1. Ge, Y., Zou, T., Martín, A. J. & Pérez-Ramírez, J. ZrO_2-promoted Cu-Co, Cu-Fe and Co-Fe catalysts for higher alcohol synthesis. *ACS Catal.* **13**, 9946–9959 (2023).
2. Toyao, T. *et al.* Machine learning for catalysis informatics: recent applications and prospects. *ACS Catal.* **10**, 2260–2297 (2020).
3. Wang, G. *et al.* Accelerated discovery of multi-elemental reverse water-gas shift catalysts using extrapolative machine learning approach. *Nat. Commun.* **14**, 5861 (2023).
4. Subramani, V. & Gangwal, S. K. A review of recent literature to search for an efficient catalytic process for the conversion of syngas to ethanol. *Energy Fuels* **22**, 814–839 (2008).
5. Spivey, J. J. & Egbebi, A. Heterogeneous catalytic synthesis of ethanol from biomass-derived syngas. *Chem. Soc. Rev.* **36**, 1514–1528 (2007).
6. Xiaoding, X., Doesburg, E. B. M. & Scholten, J. J. F. Synthesis of higher alcohols from syngas - recently patented catalysts and tentative ideas on the mechanism. *Catal. Today* **2**, 125–170 (1987).
7. Peng, J. *et al.* Human- and machine-centred designs of molecules and materials for sustainability and decarbonization. *Nat. Rev. Mater.* **7**, 991–1009 (2022).
8. Schrier, J., Norquist, A. J., Buonassisi, T. & Brgoch, J. In pursuit of the exceptional: research directions for machine learning in chemical and materials science. *J. Am. Chem. Soc.* **145**, 21699–21716 (2023).
9. Gerber, M. A., White, J. F., Gray, M. J. & Stevens, D. J. *Evaluation of Promoters for Rhodium-Based Catalysts for Mixed Alcohol Synthesis*. https://www.osti.gov/biblio/944506-bRs8yJ/ (2008) doi:10.2172/944506.
10. Gerber, M. A. *et al. Optimization of Rhodium-Based Catalysts for Mixed Alcohol Synthesis*. https://www.osti.gov/biblio/1089618/ (2010) doi:10.2172/1089618.
11. Nguyen, T. N. *et al.* High-throughput experimentation and catalyst informatics for oxidative coupling of methane. *ACS Catal.* **10**, 921–932 (2020).

12. Ramirez, A. *et al.* Accelerated exploration of heterogeneous CO_2 hydrogenation catalysts by Bayesian-optimized high-throughput and automated experimentation. *Chem Catal.* 100888 (2024) doi:10.1016/j.checat.2023.100888.
13. Sacchi, R. *et al.* PRospective EnvironMental Impact asSEment (premise): A streamlined approach to producing databases for prospective life cycle assessment using integrated assessment models. *Renew. Sustain. Energy Rev.* **160**, 112311 (2022).
14. Wernet, G. *et al.* The ecoinvent database version 3 (part I): overview and methodology. *Int. J. Life Cycle Assess.* **21**, 1218–1230 (2016).
15. Stehfest, E., van Vuuren, D., Bouwman, L. & Kram, T. Integrated assessment of global environmental change with IMAGE 3.0: model description and policy applications. https://dspace.library.uu.nl/handle/1874/308545 (2014).
16. Electricity prices around the world. *GlobalPetrolPrices.com* https://www.globalpetrolprices.com/electricity_prices/.
17. Fastepo.com. Comparison of salary of PhD students in Europe. *Fastepo* https://fastepo.com/comparison-of-salary-of-phd-students-in-europe/ (2017).
18. Zeng, Z. *et al.* A monodisperse ε′-$(Co_xFe_{1-x})_{2.2}C$ bimetallic carbide catalyst for direct conversion of syngas to higher alcohols. *ACS Catal.* **12**, 6016–6028 (2022).
19. Xu, J. *et al.* Highly selective production of long-chain aldehydes, ketones or alcohols via syngas at a mild condition. *Appl. Catal. B Environ. Energy* **307**, 121155 (2022).
